# A Functional Characterisation of a Wide Range of Cover Crop Species: Growth and Nitrogen Acquisition Rates, Leaf Traits and Ecological Strategies

**DOI:** 10.1371/journal.pone.0122156

**Published:** 2015-03-19

**Authors:** Hélène Tribouillois, Florian Fort, Pablo Cruz, Raphaël Charles, Olivier Flores, Eric Garnier, Eric Justes

**Affiliations:** 1 INRA, UMR AGIR, 24 Chemin de Borde Rouge – Auzeville, CS 52627, 31326 Castanet-Tolosan Cedex, France; 2 Agroscope, Institute of Plant Production Sciences, 50 Route de Duillier, CP 1012, CH-1260 Nyon 1, Switzerland; 3 Université de la Réunion/CIRAD, UMR—Peuplements Végétaux et Bioagresseurs en Milieu Tropical, 97410 Saint Pierre, France; 4 Centre d’Ecologie Fonctionnelle et Evolutive (UMR 5175), CNRS - Université de Montpellier - Université Paul-Valéry Montpellier – EPHE, 1919, route de Mende, 34293 Montpellier Cedex 5, France; Universidade Federal de Viçosa, BRAZIL

## Abstract

Cover crops can produce ecosystem services during the fallow period, as reducing nitrate leaching and producing green manure. Crop growth rate (CGR) and crop nitrogen acquisition rate (CNR) can be used as two indicators of the ability of cover crops to produce these services in agrosystems. We used leaf functional traits to characterise the growth strategies of 36 cover crops as an approach to assess their ability to grow and acquire N rapidly. We measured specific leaf area (SLA), leaf dry matter content (LDMC), leaf nitrogen content (LNC) and leaf area (LA) and we evaluated their relevance to characterise CGR and CNR. Cover crop species were positioned along the Leaf Economics Spectrum (LES), the SLA-LDMC plane, and the CSR triangle of plant strategies. LA was positively correlated with CGR and CNR, while LDMC was negatively correlated with CNR. All cover crops could be classified as resource-acquisitive species from their relative position on the LES and the SLA-LDMC plane. Most cover crops were located along the Competition/Ruderality axis in the CSR triangle. In particular, Brassicaceae species were classified as very competitive, which was consistent with their high CGR and CNR. Leaf functional traits, especially LA and LDMC, allowed to differentiate some cover crops strategies related to their ability to grow and acquire N. LDMC was lower and LNC was higher in cover crop than in wild species, pointing to an efficient acquisitive syndrome in the former, corresponding to the high resource availability found in agrosystems. Combining several leaf traits explained approximately half of the CGR and CNR variances, which might be considered insufficient to precisely characterise and rank cover crop species for agronomic purposes. We hypothesised that may be the consequence of domestication process, which has reduced the range of plant strategies and modified the leaf trait syndrome in cultivated species.

## Introduction

Cover crops are defined as plant covers in agrosystems whose ecosystem services reduce negative environmental impacts of agriculture, e.g. avoid groundwater nitrate pollution, protects soils against erosion or improve their fertility. Currently, cover crops are sown to i) reduce nitrate leaching by maximising nitrogen (N) acquisition before the drainage period (i.e. the “nitrate catch crop effect”) [1,2]; ii) produce “green manure” by N recycling for later release to the subsequent cash crop [3]; iii) produce biomass, which is important for soil carbon sequestration; and iv) reduce weed growth and soil seed-bank formation during the fallow period of “bare soil” between two main cash crops [1,4]. The amount of N available for the subsequent cash crop is influenced by the amount of N acquired by the cover crop, the residue quality and its decomposition rate [4]. Biomass production and N acquisition determine the C:N ratio of cover crop residues which controls the dynamics of N release from residues incorporated into the soil and then the green manure effect [5,6]. Despite the current strong interest in cover crops in cropping systems only little scientific-based information is available to choose cover crop species in a given cash crop succession on objective and sound bases. To our knowledge, only few scientific studies have been conducted to propose a method to select cover crop according to the ecosystem functions targeted. An example is the study of Damour et al. [7] which deals with tropical cover crops in banana. Traditional approaches used in agronomy are based on field experiments involving labour and time-consuming measurements (i.e. aerial and root biomass, nutrient acquisition). Since such approaches are intractable when screening a large number of species is required, we assessed whether a trait-based approach widely used in plant ecology [8–10] could be relevant for characterising the behaviour of cover crops species. We focused on leaf functional traits because they are robust, easily and quickly measurable on a wide range of species and environments.

Ecological theories [11,12] and empirical works [13,14] showed that, in wild species, relative growth rate (RGR) is related to various leaf functional traits [15,16] and it is one of the components underlying plant strategies [11,12]. Ecologists have established the existence of a fundamental trade-off between traits allowing rapid resource capture and efficient resource conservation. Specific leaf area (SLA) and leaf N concentration (LNC) appear to be effective predictors of a species’ position along the “Leaf Economic Spectrum” (LES) that captures the gradient from “acquisitive” (high SLA and LNC) to “conservative” (low SLA and LNC) species [17]. As proposed by Wilson et al. [18], leaf dry matter content (LDMC) is complementary to SLA for assessing plant functioning and resource acquisition/conservation strategies. Indeed acquisitive strategies are associated with high SLA and low LDMC whereas conservative strategies are associated with low SLA and high LDMC [19–21]. Moreover, LDMC is more strongly related to litter decomposition than SLA: litters with high LDMC tend to decompose more slowly than litters with low LDMC, leading to litter accumulation [22], thereby potentially delaying N release and then the green manure effect from cover crop residues. As emphasised above, another important potential service attributed to cover crops is limiting weed proliferation by a rapid growth, soil coverage and N acquisition, inducing a strong competition for resources which relates to their competitive abilities. Grime (1977) identified three main avenues for species’ adaptive specialisations that reflect trade-offs between their capacities to be competitive (C), stress tolerant (S) and ruderal (R). Locating species within the CSR space, and thereby assessing their relative competitive ability, normally requires measuring a large number of traits [13,23]. However, Pierce et al. [24] recently developed a new method that uses only three leaf traits-SLA, LDMC and leaf area (LA)- to assess a species’ position in the CSR space.

The aim of our study was to assess whether conceptual and methodological developments of the trait-based approach to plant functioning initially developed for wild species in natural ecosystems, could be used to characterise and classify the resource-use and ecological strategies of domesticated species such as cover crops. Species from various botanical families were analysed all together as recommend by Diaz et al. [25]. We made the hypothesis that these ecological strategies could be assessed at the cover level for cultivated species in monocrop. We hypothesised that, over cover crop species, a range of resource acquisition/conservation strategies corresponding to a range of growth and N acquisition rates could be investigated using leaf functional traits. It can be assumed that species with acquisitive strategies having high SLA and low LDMC would ensure rapid development of plant growth, N acquisition and soil covering after summer sowing, while conservative species having low SLA and high LDMC would ensure slower growth and N acquisition during and after the fallow period.

To test these hypotheses, we performed a functional characterisation of a range of cover crop using i) leaf functional traits and ii) agronomical indicators to evaluate growth and N acquisition of species such as the crop growth rate (CGR) and the crop N acquisition rate (CNR). The quantity of biomass produced during the growing period strongly depends on the CGR and on the amount of N acquired by the plants depends on the CNR [16,26]. We assumed that CGR and CNR are two relevant complementary indicators reflecting the ability of cover crops to function as a nitrate catch crop and a producer of green manure for the subsequent cash crop [1,2]. Leaf traits were measured at two experimental locations to test the sensitivity of the leaf functional trait approach to differences in environmental conditions (soils and climates in particular). To better understand where these cover crop species lie in previously identified axes of resource-use strategy variation [27] and verify the robustness of the method, these experimental data were compared to those from two large published data sets established for a large number of wild species (S1 Text): (i) one corresponding to the well-known Leaf Economics Spectrum [17] and (ii) the second one describing the SLA and LDMC relationship corresponding to the trade-off between acquisitive and conservative resource [28]. Finally, we used the methodology of Pierce et al. [24] to locate the cover crop species on the CSR space [11] and characterize differences in their ecological strategies.

## Materials and Methods

### Growth conditions

Two field experiments were conducted from August to October 2012 on research public areas at the INRA site in Auzeville-Tolosane (43°31’ N, 1°30’ E), southwest France and the Agroscope site in Nyon (46°22’ N, 6°14’ E), Switzerland. This study was permitted by the both institutions of INRA and Agroscope and field studies did not involve endangered or protected species. The French and Swiss sites had significant different mean temperatures of 18.4°C and 16.7°C during this period, respectively, and both have deep clay loam soils. Species were sown in monocrops at their recommended plant density and to constitute homogeneous dense plant covers, in plots of 10 m^2^ on 3^rd^ August 2012 in Switzerland and on 16^th^ August 2012 in France. We assumed that the two experiments conducted in non-limiting water and N availability in France and Switzerland constitute a true complete fully randomized design with two replicates corresponding to the two environmental conditions. Nonetheless, we took care to obtain very homogeneous plant covers in order to have the same range of plant intraspecific competitions for each species. Moreover, because species were grown in non-water- and non-N-limiting conditions, as proposed by Grime & Hunt (1975) [29], we can assume that these conditions allowed to both i) minimize “genotype x environment interactions” and ii) compare the potential abilities of species independently of environmental conditions. Irrigation was performed regularly and 100 kg ha^-1^ of N from fertilizer was provided to non-N fixing species at sowing. It was assumed that the symbiotic fixation of N_2_ of Fabaceae was effective thanks to the presence in soils of efficient Rhyzobium and observed good nodulation in roots for all legumes which insured optimal growth conditions and N acquisition.

### Species characteristics, crop growth and N acquisition rates

Thirty-six taxa (34 species and two varieties of *Vicia faba* and *Pisum sativum*) were selected as cover crop species based on their supposed ability to provide ecosystem services during fallow period [1,4,30]. These species are adapted to grow in French and European soils and climates and match the range of species commonly used as cover crops by farmers. Most of these species are not used as main crop in current arable cropping systems and are non-host species for pests and diseases of the main cash crops. The species were also chosen to represent a diversity of botanical families: Fabaceae, Brassicaceae, Poaceae, Asteraceae, Polygonaceae and Hydrophyllaceae. Species from the Fabaceae family are commonly used to provide green manure effect whereas Brassicaceae or Poaceae are more often used to catch nitrate from soil [3,4]. These taxa were also chosen to ensure a wide range of growth and N acquisition rates, phenology, shoot architecture and taxonomic diversity. All species had an annual life cycle except four biannual or triennial grassland species: *Secale multicaule*, *Medicago lupulina*, *Melilotus officinalis* and *Onobrychis viciifolia*. The Poaceae family was separated into two groups according to photosynthetic pathway (C_3_ versus C_4_).

We measured CGR and CNR as two complementary simple indicators-conversely to relative growth rate or RGR which is more complex to measure- to estimate plant abilities to growth and acquire N rapidly of a wide range of different species and at a single sampling date (corresponding to approximately the middle of the vegetative phase of all cover crop species). Furthermore, we measured these two indicators at the cover level, and not at the plant level, in order to take into account, in actual agronomical conditions, intraspecific competitions between plants for abiotic resources acquisition. The calculation of CGR makes it possible to compare homogeneous covers all species over a given interval of thermal time, as used in some agronomic studies [31,32]. Degree-days (DD), defined as the sum of degrees by which each days’ mean air temperature was higher than the base temperature, were calculated from the date of sowing (T0) until the date of the sampling (T1) (12 October and 28 September 2012 for the French and Swiss sites, respectively). We assumed a same base of temperature of 0°C for all species because the growth occurred in summer and early autumn, when the minimum temperature remains higher than 10°C, a temperature probably higher that the base temperature of all species. At T1, DD equalled 1160 DD and 1118 DD in the French and Swiss sites, respectively, allowing us to consider the two sites as two true statistical replicates.

Finally, CGR (in kg DM ha^-1^ DD^-1^) and CNR (in g N ha^-1^ DD^-1^) were calculated for each species at both sites using the following equations:

$$CGR=\frac{DM1-DM0}{T1-T0}\;and\;CNR=\frac{Nacq1-Nacq0}{T1-T0}$$

where DM1 and DM0 are shoot dry matters (DM) (kg ha^-1^) and Nacq1 and Nacq0 are the amounts of N acquired by plants in shoots (g ha^-1^) at T1 and T0, respectively. DM0 and Nacq0 are equal to 0 because they correspond to DM and Nacq at the sowing date. For each site and species, DM was harvested on one square meter and measured on two independent samples. N acquired was estimated by calculating total shoot N content as shoot biomass (DM) x total-shoot N concentration measured at T1 by elemental analyses based on the Dumas method (Elementar MicroVario Cube, Germany).

### Measurement of leaf traits

At each experimental site, leaf traits were measured at the vegetative stage [33] on 20 individual plants per species. Immediately after harvest, the 20 plant samples were placed in demineralised water and stored in the dark at 4°C for at least seven hours. Water was regularly sprayed on leaves to ensure that they were water-saturated [28,33]. Traits were measured on the youngest mature leaf (lamina and petiole) free from herbivore or pathogen damage. Leaves were weighed to obtain the water-saturated leaf mass and scanned with a manual scanner (Scanjet 3770, Hewlett-Packard) to measure their surface area. Leaves were then oven-dried at 60°C for 48h and weighed to measure their dry mass. Finally, leaves were ground and total-N content was measured.

LDMC was calculated as the ratio of dry mass (mg) of a leaf to its water-saturated fresh mass (g) and is expressed in mg g^-1^. SLA was calculated as LA (m^2^) divided by its dry mass (kg) and is expressed in m^2^ kg^-1^. LNC was calculated as the total amount of N (mg) per unit leaf dry mass (g) and is expressed in mg g^-1^.

### Data analysis

Co-variations among traits were evaluated using principal component analysis (PCA). We assessed the position of cover crop species along the LES axis of variation using the relationship between SLA and LNC established by Wright et al. [17]. We did the same for the SLA-LDMC relationship established for 1392 wild herbaceous species based on a compilation of published data (S1 Text). The position of species on the CSR triangle was assessed with ternary coordinates calculated from LA, LDMC and SLA values, as described by Pierce et al. [24]. Predictive regressions of this method translated PCA coordinates in multidimensional space into coordinates in a strategy triangle.

As a first analysis of the possible effect of domestication and breeding on species traits, we compared the trait values measured on cover crops at the experimental sites to those for annual wild species of the same genus available in the TRY database (Kattge et al. 2011 [34] and S2 Text). Leaf traits were extracted from the database for all native species of the same genera as those used in our experiment. For each trait, the mean value for all species within the wild genus was calculated and compared to the values obtained for the corresponding genus in our experiment. On average, four congeneric wild species were available within a wild genus for that comparison. For buckwheat (*Polygonum fagopyrum* or *Fagopyrum esculentum*), we used the wild species from the *Polygonum* genus.

Statistical analyses, correlations and generalized linear modelling (GLM) with stepwise regression procedure (stepAIC, *P*<0.05) were performed using the statistical software package R (version 2.14.0).

## Results

### Trait values and rankings are maintained between the two sites

There were no significant differences between the mean values of the four leaf traits measured in France and Switzerland (Student’s t-tests *P*-values: 0.10 for LNC, 0.42 for LDMC, 0.89 for LA and 0.90 for SLA). Consequently, species ranking based on these traits did not differ between the two sites: Spearman correlation coefficients between values obtained in France and Switzerland were 0.93, 0.87, 0.77 and 0.91 (*P*<0.0001) for SLA, LDMC, LNC and LA, respectively. Similar results were observed for CGR and CNR: there was no significant difference either in mean values (*P* = 0.23 and 0.21 respectively) or in species ranking between both sites, with Spearman correlation coefficients of 0.67 (*P* = 0.0001) and 0.62 (*P* = 0.0003), respectively.

### Species’ functional characterisation and growth and N acquisition rates

CGR and CNR varied respectively from 0.50 kg DM ha^-1^ DD^-1^ and 12.4 g N ha^-1^ DD^-1^ for *Medicago lupulina* to 5.66 kg DM ha^-1^ DD^-1^ and 155.8 g N ha^-1^ DD^-1^ for *Helianthus annuus*. Overall, Brassicaceae and Asteraceae had high CGR and CNR values, except *Camelina sativa* which had lower values (Table 1). CGR (in kg DM ha^-1^ DD^-1^) were significantly positively correlated (R^2^ = 0.73 and *P*<0.0001) with CNR (in g N ha^-1^ DD^-1^) using a logarithmic regression model (data not shown).

**Table 1:** Botanical family, taxonomic status, leaf functional traits values, crop growth rate (CGR) and crop N acquisition rate (CNR) of the 36 taxa studied.

Botanical family	Species	Id.	SLA (m^2^ kg^-1^)	LDMC (mg g^-1^)	LNC (mg g^-1^)	LA(cm²)	CGR (kg DM ha^-1^ DD^-1^)	CNR (g N ha^-1^ DD^-1^)
Asteraceae	*Guizotia abyssinica*	GA	38.2 ± 6.5	121.6 ± 12.9	41.4 ± 1.3	58.1 ± 20.4	3.43 ± 0.96	128.6 ± 32.8
	*Helianthus annuus*	HA	25.4 ± 2.4	115.9 ± 6.8	45.9 ± 3.5	222.8 ± 47.3	5.66 ± 0.52	152.8 ± 10.2
Brasicaceae	*Brassica carinata*	BC	20.1 ± 2.6	105.8 ± 8.5	39.2 ± 5.8	132.1 ± 38.6	3.71 ± 0.47	127.4 ± 12.7
	*Brassica juncea*	BJ	24.2 ± 2.8	116.6 ± 8.9	47.7 ± 6.9	79.4 ± 31.6	4.48 ± 0.28	115.6 ± 4.3
	*Brassica napus*	BN	19.1 ± 3.1	106.8 ± 11.6	38.2 ± 2.0	169.4 ± 35.9	3.21 ± 1.07	130.2 ± 40.1
	*Brassica rapa*	BR	14.3 ± 2.6	78.9 ± 9.5	38.8 ± 3.5	222.4 ± 76.9	3.07 ± 0.39	102.9 ± 15.6
	*Camelina sativa*	CS	44.9 ± 4.7	99.7 ± 11.0	61.9 ± 3.1	10.1 ± 2.8	1.96 ± 1.26	89.7 ± 55.9
	*Eruca sativa*	ES	22.6 ± 4.3	93.2 ± 19.5	51.9 ± 14.1	48.6 ± 13.4	1.97 ± 0.48	117.6 ± 25.4
	*Raphanus sativus*	RS	17.1 ± 3.0	82.7 ± 9.2	40.5 ± 5.0	222.6 ± 83.0	3.32 ± 0.29	140.8 ± 8.7
	*Sinapis alba*	SA	26.6 ± 3.4	146.6 ± 11.0	48.1 ± 6.5	80.4 ± 24.4	4.56 ± 0.48	134.2 ± 10.8
Fabaceae	*Lathyrus sativus*	LS	40.3 ± 3.4	105.5 ± 6.1	60.2 ± 2.1	19.5 ± 6.4	2.62 ± 0.16	108.8 ± 3.8
	*Lens nigricans*	LN	47.4 ± 6.1	141.5 ± 20.4	52.5 ± 0.5	9.4 ± 1.7	1.77 ± 0.06	65.5 ± 3.8
	*Lupinus angustifolius*	LA	19.2 ± 4.8	131.4 ± 14.6	32.7 ± 9.1	6.1 ± 1.7	1.2 ± 0.36	33.6 ± 10.8
	*Medicago lupulina*	ML	26.4 ± 4.5	165.0 ± 17.9	42.0 ± 5.7	9.9 ± 2.5	0.50 ± 0.02	12.2 ± 0.2
	*Melilotus officinalis*	MO	29.2 ± 3.6	156.5 ± 10.3	58.3 ± 1.9	14.9 ± 4.7	1.41 ± 0.31	71.6 ± 14.1
	*Onobrychis viciifolia*	OV	21.2 ± 2.1	160.5 ± 13.9	38.1 ± 1.6	15.9 ± 3.6	1.36 ± 0.54	51.4 ± 19.1
	*Pisum sativum cv ASSAS*	PSA	40.4 ± 4.3	111.0 ± 12.7	58.5 ± 3.1	52.8 ± 12.3	2.60 ± 0.91	124 ± 40.3
	*Pisum sativum cv PFX*	PSP	32.0 ± 3.3	106.5 ± 13.5	56.3 ± 2.7	34.9 ± 14.5	1.61 ± 0.75	72.5 ± 32
	*Trifolium alexandrinum*	TA	32.9 ± 5.3	169.4 ± 16.1	49.8 ± 1.1	13.9 ± 3.6	2.93 ± 0.47	81.9 ± 11
	*Trifolium incarnatum*	TI	30.1 ± 3.7	101.0 ± 7.9	42.2 ± 4.6	19.3 ± 4.2	1.99 ± 0.36	83.4 ± 13.1
	*Trigonella foenum-graecum*	TF	26.3 ± 4.3	143.2 ± 17.6	25.2 ± 3.5	9.0 ± 2.8	0.90 ± 0.40	20 ± 8.4
	*Vicia benghalensis*	VB	42.7 ± 5.8	132.9 ± 18.8	60.7 ± 0.3	34.2 ± 7.6	2.41 ± 0.06	111.1 ± 0.3
	*Vicia faba cv LAURA*	VFL	35.1 ± 3.7	105.4 ± 10.0	59.9 ± 3.3	85.6 ± 18.5	2.49 ± 0.17	94.5 ± 3.9
	*Vicia faba cv SSNS*	VFS	38.2 ± 3.9	104.0 ± 6.8	58.1 ± 1.7	126.1 ± 35.7	2.74 ± 0.32	96.2 ± 8.9
	*Vicia sativa*	VS	40.5 ± 3.9	122.4 ± 13.4	58.1 ± 4.5	26.8 ± 5.1	2.03 ± 0.58	59.5 ± 15.4
	*Vicia villosa*	VV	52.2 ± 6.2	120.4 ± 15.1	63.4 ± 2.4	21.3 ± 5.7	2.63 ± 0.51	124.2 ± 21.1
Hydrophyllaceae	*Phacelia tanacetifolia*	PT	35.6 ± 5.6	91.7 ± 12.2	55.5 ± 6.7	68.5 ± 25	2.56 ± 1.26	137.5 ± 64.6
Poaceae C3	*Avena sativa*	ASa	28.2 ± 3.9	146.3 ± 16.8	45.2 ± 3.4	46.1 ± 9.7	2.18 ± 0.86	72.7 ± 30.4
	*Avena strigosa*	ASt	35.2 ± 7.0	143.9 ± 21.8	47.7 ± 5.2	31.9 ± 7.2	1.60 ± 0.85	65.4 ± 36.2
	*Lolium hybridum*	LH	36.3 ± 4.0	118.5 ± 10.0	40.5 ± 5.0	22.8 ± 5.2	2.21 ± 0.51	83.6 ± 21.2
	*Lolium multiflorum*	LM	41.6 ± 5.6	132.3 ± 11.2	44.3 ± 1.7	18.5 ± 4.3	1.93 ± 0.36	77.5 ± 12.3
	*Secale cereale*	SC	32 ± 4.0	140.9 ± 14.7	51.4 ± 4.3	21.1 ± 12.2	0.91 ± 1.09	47.5 ± 57.5
	*Secale multicaule*	SM	37.5 ± 5.4	139.4 ± 15.8	48.1 ± 5.0	25.0 ± 5.7	2.01 ± 0.53	98.2 ± 23.5
Poaceae C4	*Setaria italica*	SI	28.2 ± 3.2	195.3 ± 19.1	57.4 ± 3.1	43.5 ± 22.5	1.74 ± 0.59	58.6 ± 18.3
	*Sorghum bicolor var*. *sudanense*	SS	27.8 ± 2.4	209.6 ± 28.2	35.7 ± 1.6	145.5 ± 29	3.50 ± 1.40	85.8 ± 36.3
Polygonaceae	*Polygonum fagopyrum/Fagopyrum esculentum*	PF	37.8 ± 7.4	131.7 ± 18.5	39.8 ± 6.3	27.0 ± 6.3	4.32 ± 0.10	98.9 ± 0.2

Values are means ± SDs of both experimental sites. DM is dry matter and DD is degree day.

Leaf traits also spanned a wide range of values: SLA varied from 14.3 m² kg^-1^ for *Brassica rapa* to 52.2 m^2^ kg^-1^ for *Vicia villosa*, with a mean of 31.9 m^2^ kg^-1^. LDMC varied from 78.9 mg g^-1^ for *Brassica rapa* to 209.6 mg g^-1^ for *Sorghum bicolor var*. *sudanense*, with a mean of 127.7 mg g^-1^. Although all species were grown under non-limiting N conditions, LNC also varied over a wide range, from 25.2 mg g^-1^ for *Trigonella foenum-graecum* to 63.4 mg g^-1^ for *Vicia villosa*. Finally, LA varied from 6.1 cm^2^ for *Lupinus angustifolius* to 222.8 cm^2^ for *Helianthus annuus* (Table 1). All families with more than two species (i.e. Brassicaceae, Fabaceae and Poaceae) had large variability in traits values.

### Co-variations among plant traits

**Fig 1:**
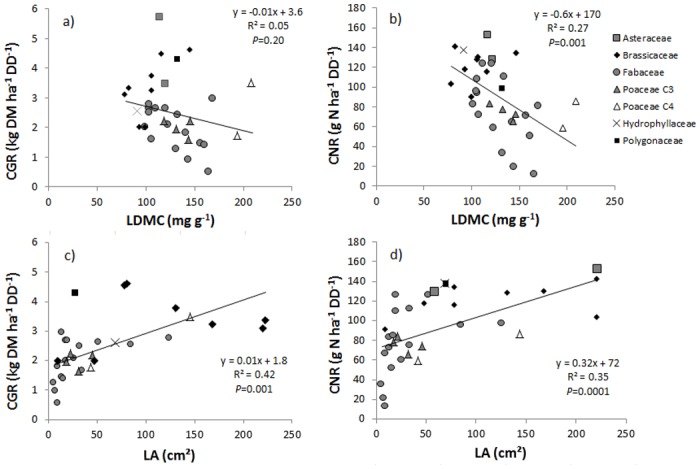
Relationships between crop growth rate (CGR) and leaf functional trait values: (a) leaf dry matter content (LDMC), (b) leaf area (LA); and relationships between crop N acquisition rate (CNR) and leaf functional trait values: (c) leaf dry matter content (LDMC), (c) leaf area (LA). The significance of differences was assessed by Student’s t-tests. Values of traits and CGRs are means of both experimental sites.

**Fig 2:**
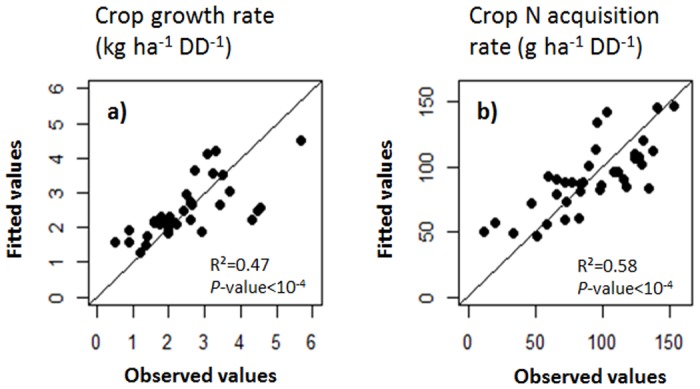
Generalized linear models based on significant leaf traits (*P*<0.05) as input variables to fit a) crop growth rate (CGR) and b) crop nitrogen acquisition rate (CNR). The equation of the CGR model is: *CGR = 4*.*8* ^*E-2*^ *SLA + 1.*5* ^*E-2*^ * LA and that for CNR is: *CNR = 63 + 0*.4*LA+ 1.5*SLA − *0*.3*LDMC, where LDMC is leaf dry matter content (mg g^-1^), LA is leaf area (cm^2^), and SLA is specific leaf area (m^2^ kg^-1^).

No significant correlation was detected between CGR and SLA, LDMC or LNC, the only significant positive relationship was found between LA and CGR, but the correlation was relatively weak (R^2^ = 0.42; *P*<0.001) (Table 2 and Fig. 1). For CNR, significant positive relationships were found but also relatively weak with LA (R^2^ = 0.35; *P*<0.001) and also with LDMC (R^2^ = 0.27; *P* = 0.001) (Table 2 and Fig. 1). These significant relationships were also obtained independently for both experimental sites (Table 2). The generalized linear models combining several leaf traits increased significantly R^2^ until 0.47 for CGR and 0.58 for CNR (Fig. 2a-b) compared to simple correlations.

**Table 2:** Pearson’s correlations between crop growth rate (CGR) and crop N acquisition (CNR) of cover crop species (n = 36) and leaf functional traits: specific leaf area (SLA), leaf dry matter content (LDMC), leaf nitrogen content (LNC) and leaf area (LA).

Experimental sites	Leaf traits	CGR (kg DM ha^-1^ DD^-1^)		CNR (g N ha^-1^ DD^-1^)	
		Corr.	*P*-value	Corr.	*P*-value
France	SLA	-0.18	0.30	-0.06	0.74
	LDMC	-0.24	0.17	-0.42	0.01
	LNC	-0.13	0.47	0.10	0.56
	LA	0.59	<0.001	0.64	<0.001
Switzerland	SLA	-0.06	0.72	0.06	0.73
	LDMC	-0.32	0.05	-0.56	<0.001
	LNC	0.05	0.78	0.26	0.12
	LA	0.60	<0.001	0.47	0.004
Both sites averaged	SLA	-0.13	0.44	0.02	0.90
	LDMC	-0.22	0.20	-0.52	0.001
	LNC	-0.01	0.72	0.23	0.18
	LA	0.65	<0.001	0.59	<0.001

DM is dry matter and DD is degree day.

**Fig 3:**
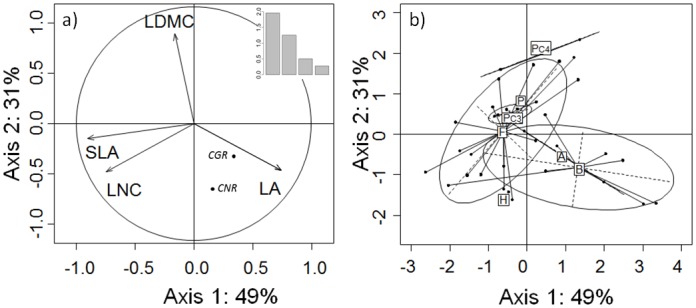
Principal component analysis (PCA) based on four functional traits measured on 36 cover crop species. (a) Correlation circle between SLA (specific leaf area), LDMC (leaf dry matter content), LNC (leaf nitrogen content) and LA (leaf area) and loadings; coordinates position of CGR (crop growth rate) and CNR (crop nitrogen acquisition rate); (b) Botanical families and cover crop species position along the first two axes of the PCA; A: Asteraceae; B: Brassicaceae; F: Fabaceae; H: Hydrophyllaceae; P: Polygonaceae; PC3: C_3_ Poaceae; PC4: C_4_ Poaceae.

The first two axes of the PCA of functional traits accounted for 80% of the variability in the data (49% and 31%, respectively). Axis 1 opposed LA to SLA and LNC (Fig. 3a). These traits strongly correlated with Axis 1 (Loading coefficients were -0.91 for SLA, -0.75 for LNC, and 0.74 for LA; all *P*<0.001). As a result, species with negative coordinates on this axis, such as *Vicia villosa*, *V*. *benghalensis*, *V*. *sativa*, *Lens nigrican*, *Camelina sativa*, had high SLA and LNC but low LA. In contrast, species with positive coordinates, such as Brassicaceae (e.g. *Brassica rapa*, *Raphanus sativus*, *B*. *napus*) had the opposite syndrome (Fig. 3b). Axis 2 was strongly and significantly positively correlated with LDMC (Pearson’s correlation coefficient was 0.89; *P*<0.001). *Sorghum bicolor var*. *sudanense*, *Trigonella foenum-graecum* and *Onobrychis viciifolia* had the highest positive coordinates on this axis, and *Brassica rapa*, *Raphanus sativus* and *Vicia faba cv* SSNS the lowest. All C_3_ Poaceae were located around the PCA centre and inside the Fabaceae ellipse, and the scatter of C_3_ Poaceae species in the former group was low. Both C_4_ Poaceae were located at a high positive position on Axis 2. Correlations between PCA axes and cover crops growth and N acquisition rates (CGR and CNR not active in PCA) were assessed (S1 Fig.): Axis 1 was significantly positively correlated with CGR but not with CNR, whereas Axis 2 was weekly negatively correlated with CGR and was more strongly correlated with CNR.

### Functional strategies of cover crop species

**Fig 4:**
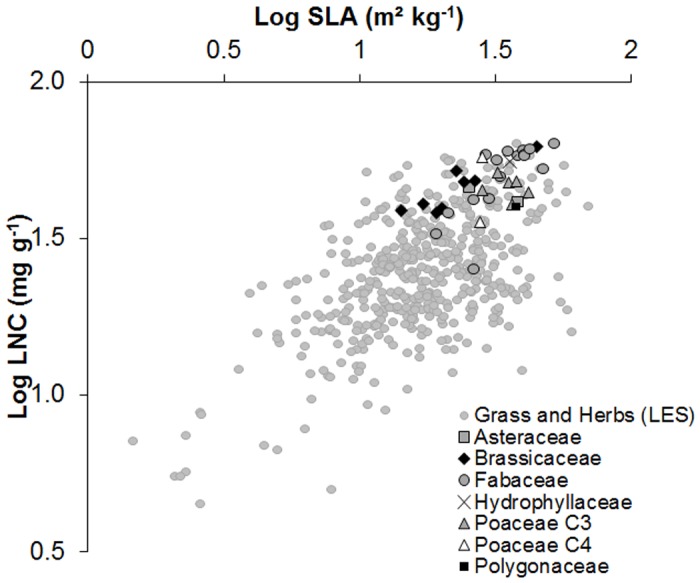
Position of cover crop species along grass and herbs of the Leaf Economic Spectrum (LES) (Wright et al. [17]) based on specific leaf area (SLA) and leaf nitrogen content (LNC) axes (the latter logarithmic).

Data from the worldwide data set used to establish general relationships involved in the leaf economics spectrum [17] show that for herbaceous species, SLA values range from 1.5 to 69.4 m^2^ kg^-1^ (mean: 19.7 m^2^ kg^-1^) and LNC values range from 4.5 to 63.6 mg g^-1^ (mean: 26.4 mg g^-1^) (Fig. 4). Cover crop species spanned 34% of the log SLA range and 35% of the log LNC range, and were located in the high SLA—high LNC zone of the LES relationship (Fig. 4). A significant positive correlation was found for cover crops species between log SLA and log LNC (R^2^ = 0.36 and *P* = 0.0001), and species from different cover crop families were not distinguishable from one another. Brassicaceae had the lowest SLA and the highest LNC, except for one species (*Camelina sativa*) (Fig. 4).

**Fig 5:**
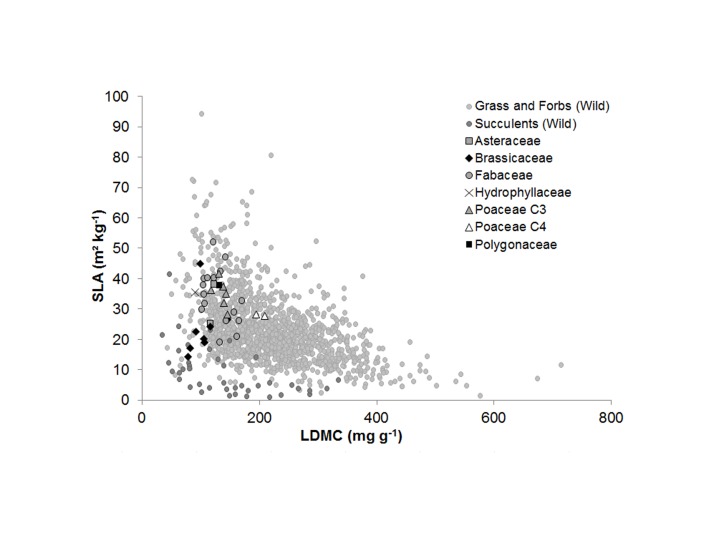
Cover crop species position compared to the relationship between leaf dry matter content (LDMC) and specific leaf area (SLA) established for 1392 grass, forbs and succulents wild species (S1 Text).

Cover crop species were positioned on the SLA-LDMC plane based on a data set composed of wild herbaceous species (Fig. 5). LDMC of wild species composed of grasses and forbs varied between 42.2 and 714 mg g^-1^ (mean: 221 mg g^-1^), while their SLA varied from 1.7–94.4 m^2^ kg^-1^ (mean: 23.2 m^2^ kg^-1^). Cover crop species spanned 48% of this range for SLA and only 25% of the LDMC range. They were mostly located in the low LDMC zone of the plane (Fig. 5), and no relationship was found between SLA and LDMC in our data set (R^2^ = 0.0006 and *P* = 0.88). Species of Fabaceae, C_3_ Poaceae, Asteraceae, Polygonaceae and Hydrophyllaceae tended to be mixed in this space. In contrast, most Brassicaceae species had relatively low SLA and LDMC and were surprisingly close to succulent species. The two C_4_ Poaceae species clearly had higher LDMC than the other cover crop species.

**Fig 6:**
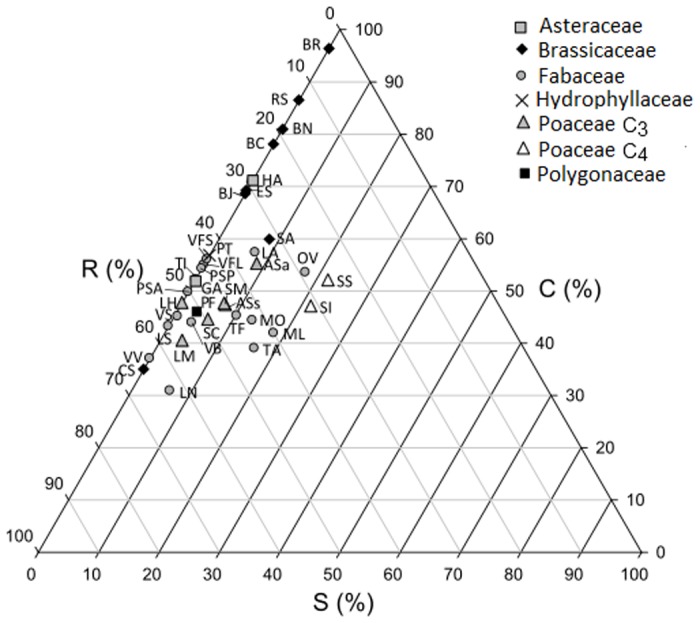
Cover crop species position on Grime’s CSR triangle (Pierce et al. [24]). C: competition, S: stress-tolerance, R: ruderality. Letters correspond to species identification (Table 1).

Cover crop strategies corresponded to a restricted region of the CSR triangle: 21 taxa were located in the C-R range in a gradient from C to R strategies (Fig. 6). All Brassicaceae were classified as competitive, with C-axis coordinates from 60–96%, except *Camelina sativa* (35% on this axis). *Helianthus annuus* (Asteraceae) was also classified as competitive, at 71% on the C-axis, as expected due to its high CGR and CNR. Species from other families were mostly positioned between competitive and ruderal (47% and 46%, on average, along the C- and R-axes, respectively). Some species were positioned as ruderal, scoring up to 65% on the R-axis (e.g. *Camelina sativa*, *Lens nigricans* and *Vicia villosa*). Species generally scored low on the stress tolerance axis, with the highest values reached by the two C_4_ Poaceae (*Sorghum bicolor var*. *sudanense* and *Setaria italica*), but with a maximum value of only 22%. Correlations between Grime’s triangle axes and CGR and CNR showed that these two indicators were positively and significantly related to the C axis (R^2^ = 0.22; *P* = 0.004 for both) but negatively and significantly related to the S axis, especially for CNR (R^2^ = 0.18; *P* = 0.050 for CGR and R^2^ = 0.35; *P*<0.001 for CNR). Both CGR and CNR were negatively related to the R axis (R^2^ = 0.11 and R^2^ = 0.04 respectively), but only the relationship with CGR was weakly significant (*P* = 0.049 and *P* = 0.223 respectively).

## Discussion

### The functional-trait approach is robust for cover crop species

Results showed that leaf functional trait values and rankings were closely maintained among cover crop species grown under non-limiting water and N availabilities at two experimental sites with different soil and climate characteristics. These results are consistent with those of Al Haj Khaled et al. [35] and Kazakou et al. [36] who demonstrated the robustness of this approach for various types of herbaceous species. In our study, in which N fertiliser was added to both sites and legumes showed good nodulation allowing efficient N_2_ fixation, LNC ranking was maintained, which might not always be the case (Kazakou et al. [36]). The results also showed that significant relationships between traits and indicators of functions (CGR and CNR) were maintained for both experimental sites. This stability in rankings, trait values and the relationships between traits and functions confirms the robustness of leaf functional traits approach for characterising functional strategies of domesticated species such as cover crops.

### Leaf traits related to cover crop growth and N acquisition rates

Axis 1 of the PCA of leaf traits showed a gradient ranging from species with small leaves, high LNC and high SLA to those with large leaves, low LNC and low SLA. This axis was positively correlated with CGR, suggesting that species with high LA, low SLA and low LNC are actually growing more quickly than those with low LA, high SLA and high LNC. However, we demonstrated that leaf area was the only leaf functional trait significantly correlated with CGR, showing that species with large leaves are growing more quickly than those with small leaves. This is consistent with the study of Hajek et al. [37] based on species used is forestry system. Contrary to our initial hypothesis, we did not find any correlation between CGR and SLA or LNC. This result suggests that, for cover crop species, there is no direct relationship between net photosynthesis expressed *per* unit leaf mass, captured by a combination of SLA and LNC [17], and the absolute growth rate of the crop cover (CGR). Leaf traits that influence growth rates of the aboveground cover (g ha^-1^ DD^-1^) therefore appear to differ from those that influence relative growth rate expressed on a plant biomass basis (RGR: g g^-1^ DD^-1^).

Axis 2 distinguished species according to their LDMC and was negatively correlated with CGR and especially with CNR. This axis indicates a gradient of species ability to acquire N (fast N uptake or high N_2_ fixation). As a result, the LDMC appeared to be the most efficient traits to estimate species ability to acquire N. Finally, the cover crop species positioned positively with axis 1 and negatively with axis 2 would be the species the most efficient as cover crops: they grow fast and acquire N rapidly after sowing and during crop establishment. This position corresponds to Brassicaceae and Asteraceae which is consistent with their highest CGR and CNR.

Finally, approximately half of the variance of CGR and CNR could be explained by a combination of several leaf functional traits. This value is relatively low and might be considered too coarse to characterize the properties of cover crop species for agronomic purposes.

### Cover crop species position along functional and ecological strategies

With their high SLA and LNC, cover crop species are located in the “acquisitive” part of the LES, which is generally associated with a high photosynthetic rate, high respiration rate and rapid leaf turnover [17]. Although the cover crop species follow the relationship between log SLA and log LNC, the LES-plane did not allow to classify them into a gradient of growth and N acquisition abilities. Although cover crops spanned a relatively large range of the SLA-LDMC distribution we did not find any significant relationship between the two traits for the cover crop species screened (cf. [28]). Brassicaceae from our data set are located close to succulents, which was not expected because of the large difference in growth rates between the two groups of species (high for Brassicaceae and low for succulents). Despite their low SLA, Brassicaceae species had high CGR and CNR. They were therefore very efficient acquisitive species that produce much biomass and are specifically sought after as cover crops adapted to short fallow periods, in relation with their early growth during autumn. However, this is consistent with their low LDMC values, showing that LMDC seems more informative than the SLA to discriminate cover crop species strategies.

We showed that positioning species on the Grime’s triangle [13] was consistent for exploring differences between cover crop species; in fact, CGR and CNR were positively correlated with the C axis. As a consequence the most competitive cover crop species are those having the highest CGR and CNR. All Brassicaceae (except *Camelina sativa*) and *Helianthus annuus* were identified as highly competitive and thus corresponds to their ability to rapidly grow and acquire N after sowing (high CGR and CNR) and occupy the space needed to acquire light. Again according to the CSR triangle, most of the cover crop species seem not to tolerate stress, except for C_4_ Poaceae. This is in agreement with the fact that C_4_ plants, especially sorghum, are often tolerant to water stress [38,39]. Cover crop species with low CGR are closer to the ruderal strategy and those with low CNR which are more stress tolerant, based on the negative correlations with R and S axes. However, we found that *Sorghum bicolor var*. *sudanense*, which should be the most stress-tolerant, had one of the highest CGR and CNR, pointing to a relative lack of precision of the method to accurately predict the strategies of cover crop species.

### A first hypothesis explaining a cover crop domestication syndrome

The hypothesis tested here is that domestication or breeding modified the values of leaf functional traits of cover crops and thus their leaf traits syndrome, i.e. the combination of leaf traits values for a given species. Examination of Fig. 5 suggests that constraints exerted by domestication have been stronger on LDMC than on SLA, as shown by the narrowest range of variation of the former trait in cover crops. We tested this hypothesis further by comparing leaf traits values of cover crops with those of their homologous wild annual species from the same genus taken from the TRY database. The analysis was done at the genus level because no data was available to confidently identify the wild ancestor of each cover crop species (Table 3). The comparison indicated that cover crop species presented systematically and significantly higher SLA and LNC but lower LDMC than wild species. Moreover, Fabaceae which did not receive any fertilizer in our experiment also presented a significant higher LNC than wild species (Table 3), suggesting that the comparison of leaf trait values between the TRY database and our experimental data is valid. This is also consistent with results found by Kazakou et al [36] who showed that SLA, LDMC and LA are not very sensitive to N fertilisation. The impact of domestication seems to have been stronger on LDMC and LNC than on SLA, based on the distance in trait values between cover crops and wild species: LDMC and LNC were respectively 37% lower and 48% higher in cover crops, while SLA was only 24% higher. This suggests domestication and breeding “forced” cover crop species towards low LDMC values, but not towards high SLA values, as also illustrated by the deformation of the SLA-LDMC relationship for cover crop species (Fig. 5). This could be related to cover crop species adaptation to different environments and reflect fundamental differences in the strategies they use to acquire, invest and use resources [40,41]. Similar results were illustrated for certain species by Gambino & Vilela (2011) [42], who found that domesticated primroses (*Primula vulgaris*) accumulated more biomass than wild ones and had higher SLA. In another study, domesticated cassava (*Manihot esculenta*) had a higher SLA and CO_2_ exchange rate and lower LDMC than wild ones [43]. Further tests of this potential differential effect of domestication on leaf trait values should be conducted on a broader range of species, with a particular emphasis on the comparison between crops and their wild ancestor.

**Table 3:** Leaf trait values of cover crop species obtained in the present study compared to trait values of congeneric wild species obtained from the TRY plant-trait database (Kattge et al. [34] and S2 Text).

	SLA (m^2^ kg^-1^)		LDMC (mg g^-1^)		LNC (mg g^-1^)	
Genera	Cover crops	Wild species	Cover crops	Wild species	Cover crops	Wild species
*Avena*	32 ± 5	27 ± 11	145 ± 2	225 ± 30	46 ± 2	26 ± 13
*Brassica*	19 ± 4	13 ± 2	102 ± 16	197 ± 14	/	na
*Camelina*	45	20 ± 3	100	149 ± 18	/	na
*Eruca*	23	12 ± 2	93	206 ± 28	/	na
*Lathyrus*	40	25 ± 11	105	206 ± 24	/	na
*Lolium*	39 ± 4	27 ± 7	125 ± 10	211 ± 47	46 ± 3	27 ± 14
*Lupinus*	/	na	/	na	33	29 ± 3
*Medicago*	26	28 ± 8	165	210 ± 62	42	38 ± 9
*Melilotus*	29	21	/	na	58	41 ± 10
*Polygonum*	38	32 ± 19	131	214 ± 56	40	20 ± 9
*Raphanus*	17	26 ± 8	83	123 ± 9	/	na
*Setaria*	28	29 ± 6	195	233 ± 45	40	18 ± 7
*Sinapis*	27	32 ± 8	147	126 ± 5	/	na
*Trifolium*	31 ± 2	27 ± 11	135 ± 48	258 ± 77	46 ± 5	29 ± 6
*Vicia*	42 ± 6	25 ± 8	117 ± 12	218 ± 36	60 ± 2	54
**Average**	31 ± 9	25 ± 5	126 ± 32	199 ± 41	46 ± 8	31 ± 11
**P-value**	0.03		<10^-4^		0.008	
**Relative distance**	**+ 24%**		**- 37%**		**+ 48%** **(+ 26% for Fabaceae)**	

Traits of wild species are means of annual species of same genus. Values are means ± SDs of species of each family (na: data not available in TRY database). The significance of differences was assessed by Student’s t-tests, testing the global effect for all genera. The relative distance was calculated as the difference between the average values of cover crops and the average values of wild species as: *Relative distance rate = (Cover crop trait value—wild species trait value) / wild species trait value*.

### Cover crop traits and ecosystem functions related to N management

Overall, the fact that LDMC of cover crops was reduced while at the same time LNC was increased in comparison to wild species indicates that cover crops are effective acquisitive species inducing rapid biomass production, ability for photosynthesis and N accumulation in their tissue. This leaf trait syndrome of cover crops illustrates their ability to provide both i) an efficient “nitrate catch crop” function by trapping rapidly most of the nitrate available in the soil (high CGR and CNR), and ii) a green manure function by increasing the amount of N acquired and accumulated in the tissues of the cover crop (high CNR). Moreover, the relatively low LDMC values of cover crops indicate that the N release for the subsequent cash crop will be favoured, since litters coming from leaves with low LDMC tend to decompose rapidly [22,44]. If so, cover crop species would tend to produce higher-quality residue, increasing the rate of N release after residue incorporation into the soil and then produce N green manure effect. Considering the decomposition of crop residues, our results seem consistent with García-Palacios et al. [45], who worked with some of the species tested in our experiments (*Avena sativa*, *Helianthus annuus*, *Secale cereale*, *Sorghum sudanense*). These authors showed that domesticated species produced a litter of higher quality than wild species of the same genus. Among the cover crop species considered in this study, *Phacelia tanacetifolia*, some Brassiceae and Fabaceae presented low LDMC suggesting that these species would be efficient to fulfil the N green manure function. In a modelling study, Justes et al. [6] demonstrated that the lower the C:N ratio and then the highest LNC, the more rapidly N is released from cover crop residues after their incorporation into the soil, reinforcing the green manure function of cover crops, in particular for legumes having high LNC and low LDMC.

## Conclusion

Leaf functional traits allowed us to explore differences in the ability of cover crop species to grow fast and rapidly acquire N after sowing and during the cover establishment. Despite the wide range of CGR and CNR spanned by the studied species, they can be considered as belonging to the acquisition-use strategy as indicated by converging results from the three ecological methods tested. We demonstrated that LA and LMDC were correlated to crop growth and N acquisition rates and that combining several leaf traits allowed us to explain approximately half of the CGR and CNR variances. This might be considered insufficient to precisely characterise and rank the cover crop species for agronomic purposes. Furthermore, this study pointed out that LDMC values of cover crops was substantially lower than those of wild species. We hypothesised that domestication and breeding would have modified the leaf trait values of cultivated species as cover crops. Further studies are necessary to better understand the links between functional traits and the functions of cover crops, so as to improve the use of trait based approach in the perspective of choosing species in relation to targeted ecosystem functions.

## Supporting Information

S1 Fig Relationships between crop growth rate (CGR) and crop N acquisition rate (CNR) and strategy axes from principal component analysis (PCA): axis 1 and 2. The significance of differences was assessed by Student’s t-tests. Values of traits and CNR and CGR are means of both experimental sites.
(TIF)

S1 Text List of references of published data used to establish the SLA-LDMC relationship on 2176 wild species. (PDF)

S2 Text List of original references via the global TRY plant trait database. (PDF)
