# Supplementary figures and images for: A Functional Characterisation of a Wide Range of Cover Crop Species: Growth and Nitrogen Acquisition Rates, Leaf Traits and Ecological Strategies
Source: PLoS One. 2015 Mar 19;10(3):e0122156. doi: 10.1371/journal.pone.0122156 (PMC4366015; doi:10.1371/journal.pone.0122156)

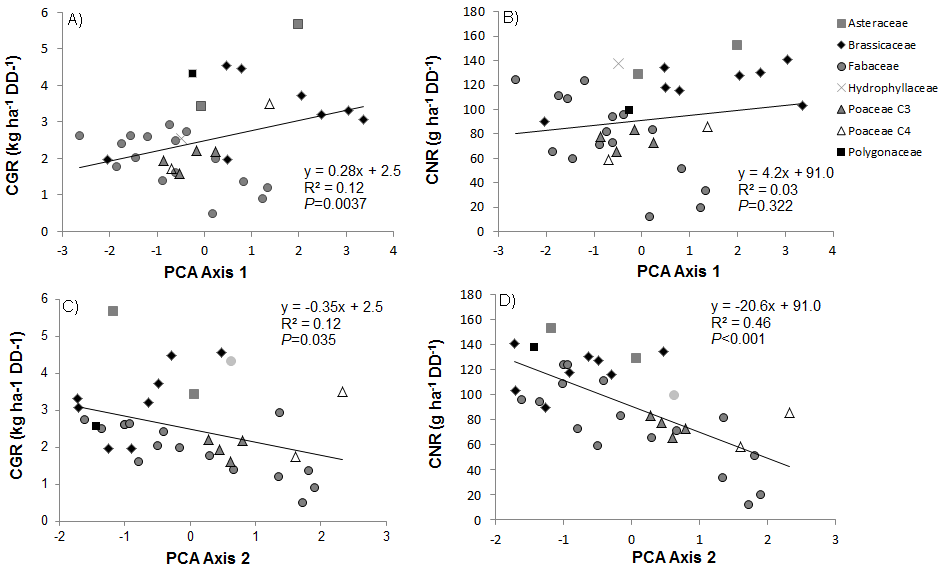

Supplement: S1 Fig — The significance of differences was assessed by Student’s t-tests. Values of traits and CNR and CGR are means of both experimental sites. (TIF) [file pone.0122156.s001.tif]
