# Supplementary material for: A Functional Characterisation of a Wide Range of Cover Crop Species: Growth and Nitrogen Acquisition Rates, Leaf Traits and Ecological Strategies
Source: PLoS One. 2015 Mar 19;10(3):e0122156. doi: 10.1371/journal.pone.0122156 (PMC4366015; doi:10.1371/journal.pone.0122156)
Supplement: S1 Text — (PDF) [file pone.0122156.s002.pdf]

Bongers, F. & Popma, J. (1990) Leaf characteristics of the tropical rain forest flora of Los Tuxtlas, Mexico. *Botanical Gazette*, **151**, 354–365.

Caccianiga, M., Luzzaro, A., Pierce, S., Ceriani, R.M. & Cerabolini, B. (2006) The functional basis of a primary succession resolved by CSR classification. *Oikos*, **112**, 10–20.

Choong, M.F., Lucas, P.W., Ong, J.S.Y., Pereira, B., Tan, H.T.W. & Turner, I.M. (1992) Leaf fracture toughness and sclerophylly: their correlations and ecological implications. *New Phytologist*, **121**, 597–610.

Cornelissen, J.H.C., Werger, M.J.A., Castro-Díez, P., van Rheenen, J.W.A. & Rowland, A.P. (1997) Foliar nutrients in relation to growth, allocation and leaf traits in seedlings of a wide range of woody plant species and types. *Oecologia*, **111**, 460.

Easdale, T.A., Gurvich, D.E., Sersic, A.N. & Healey, J.R. (2007) Tree morphology in seasonally dry montane forest in Argentina: Relationships with shade tolerance and nutrient shortage. *Journal of Vegetation Science*, **18**, 313–326.

Garnier, E., Lavorel, S., Ansquer, P., Castro, H., Cruz, P., Dolezal, J., Eriksson, O., Fortunel, C., Freitas, H., Golodets, C., Grigulis, K., Jouany, C., Kazakou, E., Kigel, J., Kleyer, M., Lehsten, V., Leps, J., Meier, T., Pakeman, R., Papadimitriou, M., Papanastasis, V.P., Quested, H., Quétier, F., Robson, M., Roumet, C., Rusch, G., Skarpe, C., Sternberg, M., Theau, J.-P., Thébault, A., Vile, D. & Zarovali, M.P. (2007) Assessing the effects of land-use change on plant traits, communities and ecosystem functioning in grasslands: a standardized methodology and lessons from an application to 11 European sites. *Annals of botany*, **99**, 967–85.

Gohel., N. (2001) Etude de la résilience de la composition spécifique et fonctionnelle en friche méditerranéenne face aux perturbations, l'hypothèse de redondance fonctionnelle est-elle vérifiée ? Master thesis. Université de Montpellier II Sciences et Techniques du Languedoc, France.

Hao, G.-Y., Hoffmann, W.A., Scholz, F.G., Bucci, S.J., Meinzer, F.C., Franco, A.C., Cao, K.-F. & Goldstein, G. (2008) Stem and leaf hydraulics of congeneric tree species from adjacent tropical savanna and forest ecosystems. *Oecologia*, **155**, 405–15.

Jayasekera, R. (1992) Elemental concentrations in a tropical montane rain forest in Sri Lanka. *Vegetatio*, **98**, 73–81.

Kleyer, M., Bekker, R.M., Knevel, I.C., Bakker, J.P., Thompson, K., Sonnenschein, M., Poschlod, P., van Groenendael, J.M., Klimeš, L., Klimešová, J., Klotz, S., Rusch, G.M., Hermy, M., Adriaens, D., Boedeltje, G., Bossuyt, B., Dannemann, A., Endels, P., Götzenberger, L., Hodgson, J.G., Jackel, A.-K., Kühn, I., Kunzmann, D., Ozinga, W.A., Römermann, C., Stadler, M., Schlegelmilch, J., Steendam, H.J., Tackenberg, O., Wilmann, B., Cornelissen, J.H.C., Eriksson, O.,

Garnier, E. & Peco, B. (2008) The LEDA Traitbase: a database of life-history traits of the Northwest European flora. *Journal of Ecology*, **96**, 1266–1274.

Lavergne, S., Garnier, E. & Debussche, M. (2003) Do rock endemic and widespread plant species differ under the Leaf-Height-Seed plant ecology strategy scheme? *Ecology Letters*, **6**, 398–404.

Mokany, K. & Ash, J. (2008) Are traits measured on pot grown plants representative of those in natural communities? *Journal of Vegetation Science,* **19**, 119–126.

Paula, S. & Pausas, J.G. (2006) Leaf traits and resprouting ability in the Mediterranean basin. *Functional Ecology*, **20**, 941–947.

Pierce, S., Brusa, G., Vagge, I. & Cerabolini, B.E.L. (2013) Allocating CSR plant functional types: the use of leaf economics and size traits to classify woody and herbaceous vascular plants (ed K Thompson). *Functional Ecology*, **27**, 1002–1010.

Pierce, S., Luzzaro, A., Caccianiga, M., Ceriani, R.M. & Cerabolini, B. (2007) Disturbance is the principal α-scale filter determining niche differentiation, coexistence and biodiversity in an alpine community. *Journal of Ecology*, **95**, 698–706.

Prior, L.D., Eamus, D. & Bowman, D.M.J.S. (2003) Leaf attributes in the seasonally dry tropics: a comparison of four habitats in northern Australia. *Functional Ecology*, **17**, 504–515.

Roderick, M.L., Berry, S.L., Saunders, A.R. & Noble, I.R. (1999) On the relationship between the composition, morphology and function of leaves. *Functional Ecology*, **13**, 696–710.

Saura-Mas, S. & Lloret, F. (2007) Leaf and shoot water content and leaf dry matter content of Mediterranean woody species with different post-fire regenerative strategies. *Annals of botany*, **99**, 545–54.

Shipley, B. (1995) Structured interspecific determinants of specific leaf area in 34 species of herbaceous angiosperms. *Functional Ecology,* **9**, 312–319.

Shipley, B. & Vu, T.-T. (2002) Dry matter content as a measure of dry matter concentration in plants and their parts. *New Phytologist*, **153**, 359–364.

Vendramini, F., Díaz, S., Gurvich, D.E., Wilson, P.J., Thompson, K. & Hodgson, J.G. (2002) Leaf traits as indicators of resource-use strategy in floras with succulent species. *New Phytologist*, **154**, 147–157.

Vile, D. (2001) Traits foliaires des espèces végétales, signification fonctionnelle et variation sur un gradient d’aridité. Master thesis. Université de Montpellier II Sciences et Techniques du Languedoc, France.

Wang, G. (2007) Leaf trait co-variation, response and effect in a chronosequence. *Journal of Vegetation Science*, **18**, 563–570.

Willert, D.J., Eller, B.M., Werger, M.J.A. & Brinckmann, E. (1990) Desert succulents and their life strategies. *Vegetatio*, **90**, 133–143.

Wilson, P.J., Thompson, K. & Hodgson, J.G. (1999) Specific leaf area and leaf dry matter content as alternative predictors of plant strategies. *New Phytologist*, **143**, 155–162.

Wright, I.J. (2001) Leaf economics of perennial species from sites contrasted on rainfall and soil nutrients. Ph.D. Thesis. Macquarie University, Australia.

Wright, I.J., Reich, P.B. & Westoby, M. (2001) Strategy shifts in leaf physiology, structure and nutrient content between species of high- and low-rainfall and high- and low-nutrient habitats. *Functional Ecology*, **15**, 423–434.

Zotz, G., Tyree, M.T., Patiño, S. & Carlton, M.R. (1998) Hydraulic architecture and water use of selected species from a lower montane forest in Panama. *Trees*, **12**, 302.
