# Supplementary material for: A Functional Characterisation of a Wide Range of Cover Crop Species: Growth and Nitrogen Acquisition Rates, Leaf Traits and Ecological Strategies
Source: PLoS One. 2015 Mar 19;10(3):e0122156. doi: 10.1371/journal.pone.0122156 (PMC4366015; doi:10.1371/journal.pone.0122156)
Supplement: S2 Text — (PDF) [file pone.0122156.s003.pdf]

Data from the TRY database used in this study come from original studies cited below:

Cornwell, W.K., Cornelissen, J.H.C., Amatangelo, K., Dorrepaal, E., Eviner, V.T., Godoy, O., Hobbie, S.E., Hoorens, B., Kurokawa, H., Pérez-Harguindeguy, N., Quested, H.M., Santiago, L.S., Wardle, D.A., Wright, I.J., Aerts, R., Allison, S.D., van Bodegom, P., Brovkin, V., Chatain, A., Callaghan, T. V, Díaz, S., Garnier, E., Gurvich, D.E., Kazakou, E., Klein, J.A., Read, J., Reich, P.B., Soudzilovskaia, N.A., Vaieretti, M.V. & Westoby, M. (2008) Plant species traits are the predominant control on litter decomposition rates within biomes worldwide. *Ecology Letters*, **11**, 1065–71.

Fonseca, C.R., Overton, J.M., Collins, B. & Westoby, M. (2000) Shifts in trait-combinations along rainfall and phosphorus gradients. *Journal of Ecology*, **88**, 964–977.

Fortunel, C., Garnier, E., Joffre, R., Kazakou, E., Quested, H., Grigulis, K., Lavorel, S., Ansquer, P., Castro, H., Cruz, P., Dolezal, J., Eriksson, O., Freitas, H., Golodets, C., Jouany, C., Kigel, J., Kleyer, M., Lehsten, V., Leps, J., Meier, T., Pakeman, R., Papadimitriou, M., Papanastasis, V.P., Quetier, F., Robson, M. & Sternberg, M. (2009a) Leaf traits capture the effects of land use changes and climate on litter decomposability of grasslands across Europe. *Ecology*, **90**, 598–611.

Fortunel, C., Garnier, E., Joffre, R., Kazakou, E., Quested, H., Grigulis, K., Lavorel, S., Ansquer, P., Castro, H., Cruz, P., Doležal, J., Eriksson, O., Freitas, H., Golodets, C., Jouany, C., Kigel, J., Kleyer, M., Lehsten, V., Lepš, J., Meier, T., Pakeman, R., Papadimitriou, M., Papanastasis, V.P., Quétier, F., Robson, M., Sternberg, M., Theau, J.-P., Thébault, A. & Zarovali, M. (2009b) Leaf traits capture the effects of land use changes and climate on litter decomposability of grasslands across Europe. *Ecology*, **90**, 598–611.

Garnier, E., Lavorel, S., Ansquer, P., Castro, H., Cruz, P., Dolezal, J., Eriksson, O., Fortunel, C., Freitas, H., Golodets, C., Grigulis, K., Jouany, C., Kazakou, E., Kigel, J., Kleyer, M., Lehsten, V., Leps, J., Meier, T., Pakeman, R., Papadimitriou, M., Papanastasis, V.P., Quested, H., Quétier, F., Robson, M., Roumet, C., Rusch, G., Skarpe, C., Sternberg, M., Theau, J.-P., Thébault, A., Vile, D. & Zarovali, M.P. (2007) Assessing the effects of land-use change on plant traits, communities and ecosystem functioning in grasslands: a standardized methodology and lessons from an application to 11 European sites. *Annals of Botany*, **99**, 967–85.

Han, W., Fang, J., Guo, D. & Zhang, Y. (2005) Leaf nitrogen and phosphorus stoichiometry across 753 terrestrial plant species in China. *The New Phytologist*, **168**, 377–85.

He, J.-S., Wang, L., Flynn, D.F.B., Wang, X., Ma, W. & Fang, J. (2008) Leaf nitrogen:phosphorus stoichiometry across Chinese grassland biomes. *Oecologia*, **155**, 301–10.

He, J.-S., Wang, Z., Wang, X., Schmid, B., Zuo, W., Zhou, M., Zheng, C., Wang, M. & Fang, J. (2006) A test of the generality of leaf trait relationships on the Tibetan Plateau. *The New Phytologist*, **170**, 835–48.

Hickler, T. (1999) Plant Functional Types and Community Characteristics along Environmental Gradients on Öland's Great Alvar (Sweden). Master Thesis. University of Lund, Sweden.

Kattge, J., Knorr, W., Raddatz, T. & Wirth, C. (2009) Quantifying photosynthetic capacity and its relationship to leaf nitrogen content for global-scale terrestrial biosphere models. *Global Change Biology*, **15**, 976–991.

Kazakou, E., Violle, C., Roumet, C., Pintor, C., Gimenez, O. & Garnier, E. (2009) Litter quality and decomposability of species from a Mediterranean succession depend on leaf traits but not on nitrogen supply. *Annals of Botany*, **104**, 1151–1161.

Kleyer, M., Bekker, R.M., Knevel, I.C., Bakker, J.P., Thompson, K., Sonnenschein, M., Poschlod, P., van Groenendael, J.M., Klimeš, L., Klimešová, J., Klotz, S., Rusch, G.M., Hermy, M., Adriaens, D., Boedeltje, G., Bossuyt, B., Dannemann, A., Endels, P., Götzenberger, L., Hodgson, J.G., Jackel, A.-K., Kühn, I., Kunzmann, D., Ozinga, W. a., Römermann, C., Stadler, M., Schlegelmilch, J., Steendam, H.J., Tackenberg, O., Wilmann, B., Cornelissen, J.H.C., Eriksson, O., Garnier, E. & Peco, B. (2008) The LEDA Traitbase: a database of life-history traits of the Northwest European flora. *Journal of Ecology*, **96**, 1266–1274.

Laughlin, D.C., Leppert, J.J., Moore, M.M. & Sieg, C.H. (2010) A multi-trait test of the leaf-height-seed plant strategy scheme with 133 species from a pine forest flora. *Functional Ecology*, **24**, 493–501.

McDonald, P.G., Fonseca, C.R., Overton, J.M. & Westoby, M. (2003) Leaf-size divergence along rainfall and soil-nutrient gradients: is the method of size reduction common among clades? *Functional Ecology*, **17**, 50–57.

McKenna, M.F. & Shipley, B. (1999) Interacting determinants of interspecific relative growth: Empirical patterns and a theoretical explanation. *Ecoscience*, **6**, 286–296.

Meziane, D. & Shipley, B. (1999a) Interacting determinants of specific leaf area in 22 herbaceous species: effects of irradiance and nutrient availability. *Plant, Cell & Environment*, **22**, 447–459.

Meziane, D. & Shipley, B. (1999b) Interacting components of interspecific relative growth rate: constancy and change under differing conditions of light and nutrient supply. *Functional Ecology*, **13**, 611–622.

Onoda, Y., Westoby, M., Adler, P.B., Choong, A.M.F., Clissold, F.J., Cornelissen, J.H.C., Díaz, S., Dominy, N.J., Elgart, A., Enrico, L., Fine, P.V.A., Howard, J.J., Jalili, A., Kitajima, K., Kurokawa, H., McArthur, C., Lucas, P.W., Markesteijn, L., Pérez-Harguindeguy, N., Poorter, L., Richards, L., Santiago, L.S., Sosinski,

E.E., Van Bael, S.A., Warton, D.I., Wright, I.J., Wright, S.J. & Yamashita, N. (2011) Global patterns of leaf mechanical properties. *Ecology Letters*, **14**, 301–12.

Ordoñez, J.C., van Bodegom, P.M., Witte, J.-P.M., Bartholomeus, R.P., van Dobben, H.F. & Aerts, R. (2010a) Leaf habit and woodiness regulate different leaf economy traits at a given nutrient supply. *Ecology*, **91**, 3218–3228.

Ordoñez, J.C., Bodegom, P.M. van, Witte, J.M., Bartholomeus, R.P., Hal, J.R. van & Aerts, R. (2010b) Plant strategies in relation to resource supply in mesic to wet environments: does theory mirror nature? *The American Naturalist*, **175**, 225–239.

Pakeman, R.J., Garnier, E., Lavorel, S., Ansquer, P., Castro, H., Cruz, P., Doležal, J., Eriksson, O., Freitas, H., Golodets, C., Kigel, J., Kleyer, M., Lepš, J., Meier, T., Papadimitriou, M., Papanastasis, V.P., Quested, H., Quétier, F., Rusch, G., Sternberg, M., Theau, J.-P., Thébault, A. & Vile, D. (2008) Impact of abundance weighting on the response of seed traits to climate and land use. *Journal of Ecology*, **96**, 355–366.

Pakeman, R.J., Lepš, J., Kleyer, M., Lavorel, S. & Garnier, E. (2009) Relative climatic, edaphic and management controls of plant functional trait signatures. *Journal of Vegetation Science*, **20**, 148–159.

Poorter, H., Niinemets, Ü., Poorter, L., Wright, I.J. & Villar, R. (2009) Causes and consequences of variation in leaf mass per area (LMA): a meta-analysis. *New Phytologist*, **182**, 565–588.

Reich, P.B., Oleksyn, J. & Wright, I.J. (2009) Leaf phosphorus influences the photosynthesis-nitrogen relation: a cross-biome analysis of 314 species. *Oecologia*, **160**, 207–12.

Reich, P.B., Tjoelker, M.G., Pregitzer, K.S., Wright, I.J., Oleksyn, J. & Machado, J.-L. (2008) Scaling of respiration to nitrogen in leaves, stems and roots of higher land plants. *Ecology Letters*, **11**, 793–801.

Shipley, B. (1995) Structured interspecific determinants of specific leaf area in 34 species of herbaceous angiosperms. *Functional Ecology*, **9**, 312–319.

Shipley, B. (2002) Trade-offs between net assimilation rate and specific leaf area in determining relative growth rate: relationship with daily irradiance. *Functional Ecology*, **16**, 682–689.

Shipley, B. & Lechowicz, M.J. (2000) The functional co-ordination of leaf morphology, nitrogen concentration, and gas exchange in 40 wetland species. *Ecoscience*, **7**, 183–194.

Shipley, B. & Vu, T.-T. (2002) Dry matter content as a measure of dry matter concentration in plants and their parts. *New Phytologist*, **153**, 359–364.

Vile, D. (2005) Significations fonctionnelle et écologique des traits des espèces végétales: exemple dans une succession post-culturale méditerranéenne et généralisations. Ph.D. Thesis. Université des sciences et techniques de Montpellier 2, France.

Willis, C.G., Halina, M., Lehman, C., Reich, P.B., Keen, A., McCarthy, S. & Cavender-Bares, J. (2009) Phylogenetic community structure in Minnesota oak savanna is influenced by spatial extent and environmental variation. *Ecography*, **33**, 565-577.

Wright, I.J., Reich, P.B., Atkin, O.K., Lusk, C.H., Tjoelker, M.G. & Westoby, M. (2006) Irradiance, temperature and rainfall influence leaf dark respiration in woody plants: evidence from comparisons across 20 sites. *The New Phytologist*, **169**, 309–19.

Wright, I.J., Reich, P.B., Westoby, M., Ackerly, D.D., Baruch, Z., Bongers, F., Cavender-Bares, J., Chapin, T., Cornelissen, J.H.C., Diemer, M., Flexas, J., Garnier, E., Groom, P.K., Gulias, J., Hikosaka, K., Lamont, B.B., Lee, T., Lee, W., Lusk, C., Midgley, J.J., Navas, M.-L., Niinemets, U., Oleksyn, J., Osada, N., Poorter, H., Poot, P., Prior, L., Pyankov, V.I., Roumet, C., Thomas, S.C., Tjoelker, M.G., Veneklaas, E.J. & Villar, R. (2004) The worldwide leaf economics spectrum. *Nature*, **428**, 821–7.
